# Supplementary material for: New Insights on the Role of ß-Cyanoalanine Synthase CAS-C1 in Root Hair Elongation through Single-Cell Proteomics
Source: Plants (Basel). 2023 Dec 2;12(23):4055. doi: 10.3390/plants12234055 (PMC10708425; doi:10.3390/plants12234055)
Supplement: Supplementary file 1 [file plants-12-04055-s001.zip › SUPPLEMENTARY MATERIAL_v3/Supplementary Figures root hair Lucía_Plants_v3.pptx]

## Slide 1
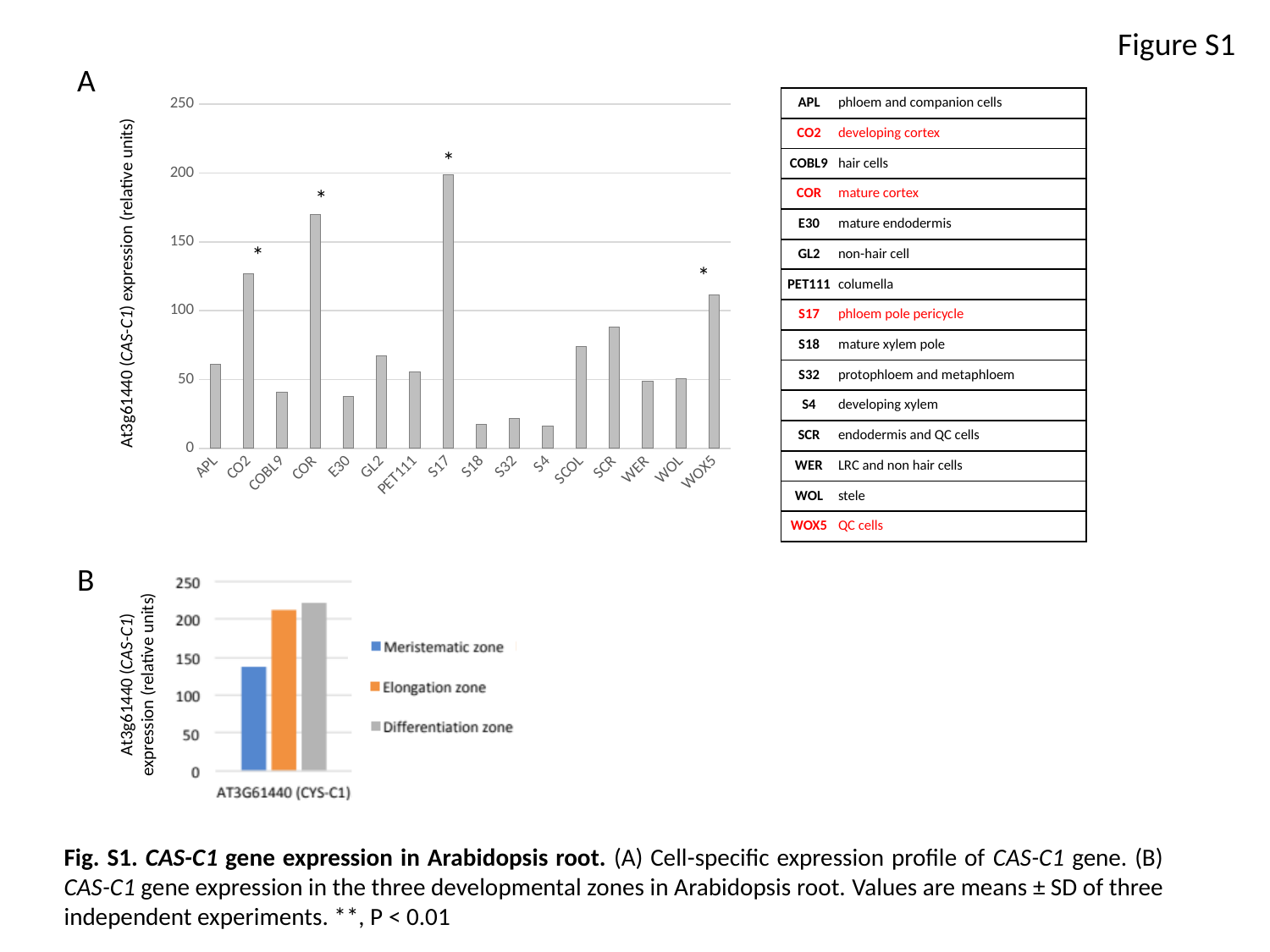

Figure S1
A
### Chart
| Category | AT3G61440 (CYS-C1) |
|---|---|
| APL | 60.8127333 |
| CO2 | 126.814333 |
| COBL9 | 40.5511333 |
| COR | 170.187 |
| E30 | 37.6376 |
| GL2 | 67.2317333 |
| PET111 | 55.3222 |
| S17 | 199.095667 |
| S18 | 17.2854 |
| S32 | 22.0214 |
| S4 | 16.2602333 |
| SCOL | 73.9021 |
| SCR | 88.3049333 |
| WER | 48.8597333 |
| WOL | 50.4535333 |
| WOX5 | 111.263 |*
*
*
*
At3g61440 (CAS-C1) expression (relative units)
| APL | phloem and companion cells |
| --- | --- |
| CO2 | developing cortex |
| COBL9 | hair cells |
| COR | mature cortex |
| E30 | mature endodermis |
| GL2 | non-hair cell |
| PET111 | columella |
| S17 | phloem pole pericycle |
| S18 | mature xylem pole |
| S32 | protophloem and metaphloem |
| S4 | developing xylem |
| SCR | endodermis and QC cells |
| WER | LRC and non hair cells |
| WOL | stele |
| WOX5 | QC cells |
B
At3g61440 (CAS-C1)
expression (relative units)
Fig. S1. CAS-C1 gene expression in Arabidopsis root. (A) Cell-specific expression profile of CAS-C1 gene. (B) CAS-C1 gene expression in the three developmental zones in Arabidopsis root. Values are means ± SD of three independent experiments. **, P ˂ 0.01

## Slide 2
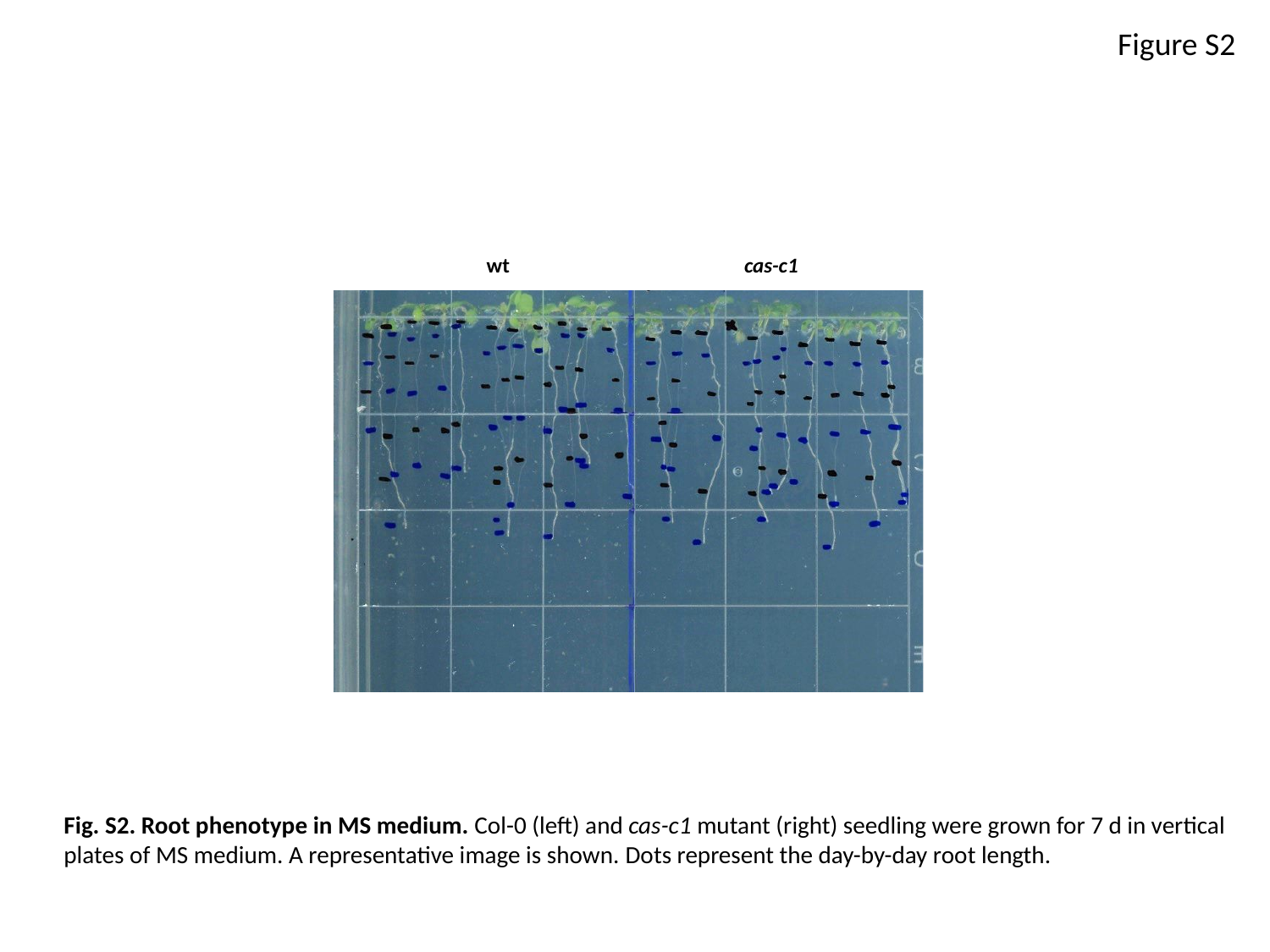

Figure S2
wt
cas-c1
Fig. S2. Root phenotype in MS medium. Col-0 (left) and cas-c1 mutant (right) seedling were grown for 7 d in vertical plates of MS medium. A representative image is shown. Dots represent the day-by-day root length.

## Slide 3
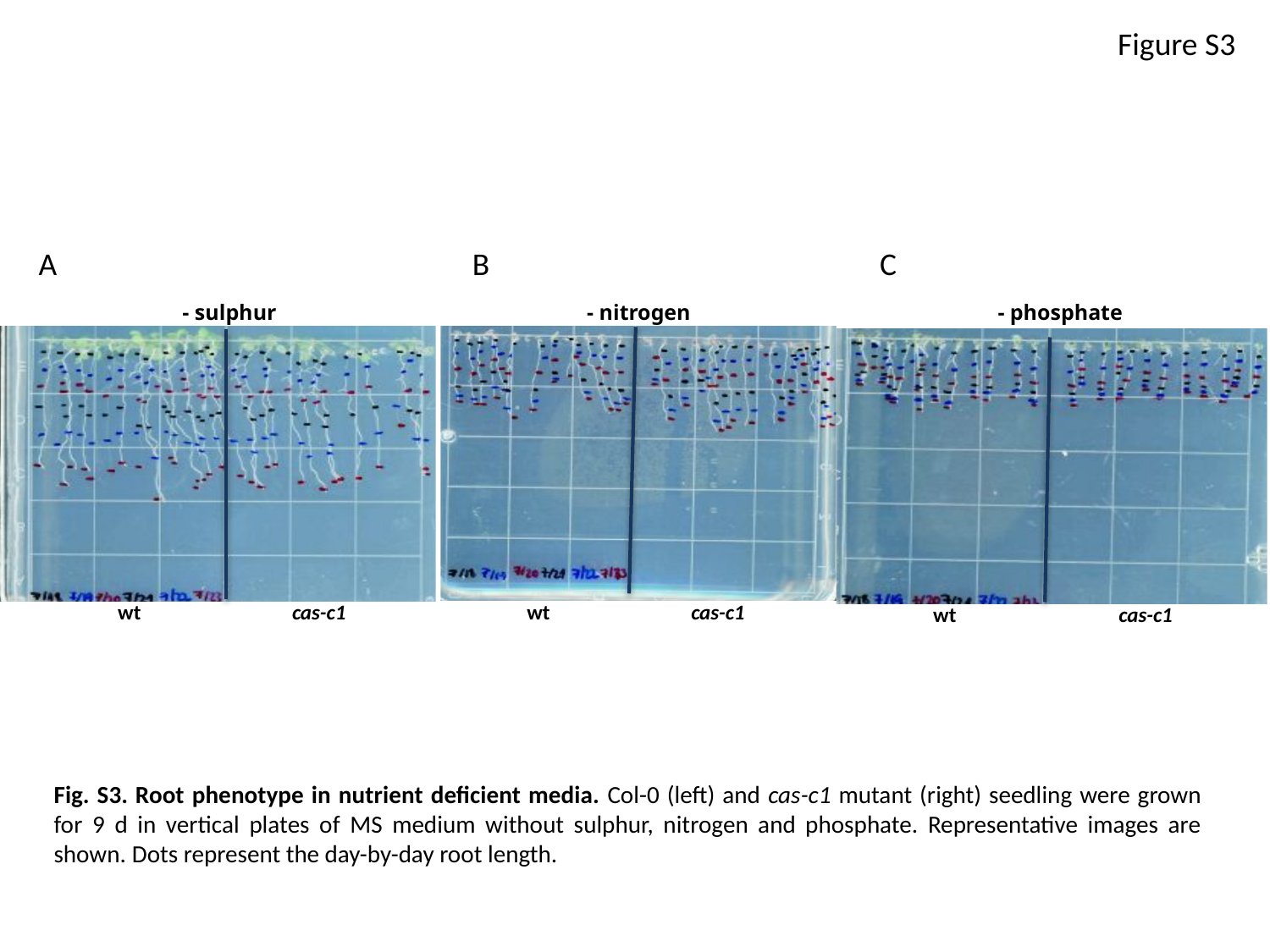

Figure S3
A
B
C
- sulphur
wt
cas-c1
- nitrogen
wt
cas-c1
- phosphate
wt
cas-c1
Fig. S3. Root phenotype in nutrient deficient media. Col-0 (left) and cas-c1 mutant (right) seedling were grown for 9 d in vertical plates of MS medium without sulphur, nitrogen and phosphate. Representative images are shown. Dots represent the day-by-day root length.

## Slide 4
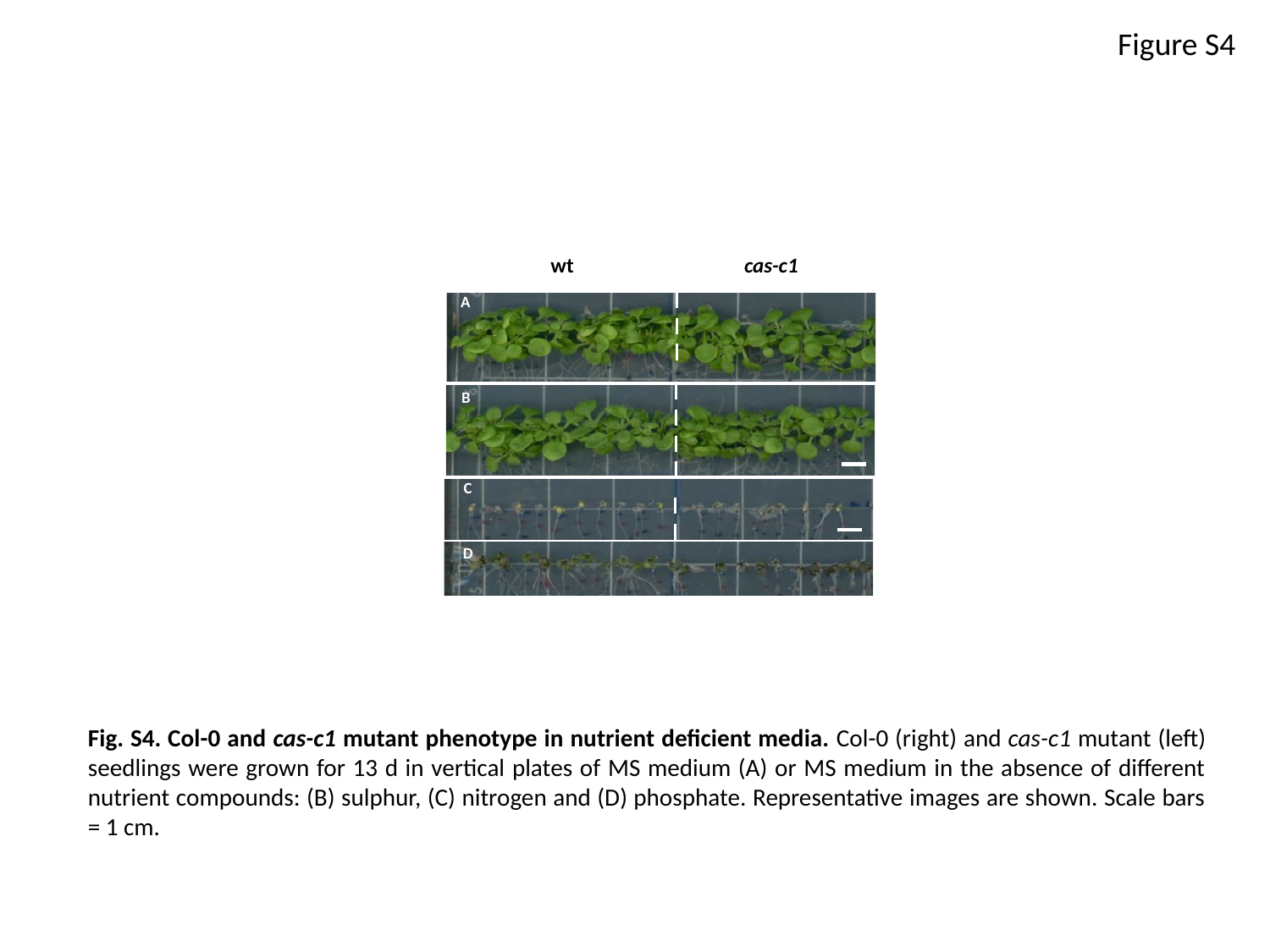

Figure S4
wt
cas-c1
A
B
C
D
D
Fig. S4. Col-0 and cas-c1 mutant phenotype in nutrient deficient media. Col-0 (right) and cas-c1 mutant (left) seedlings were grown for 13 d in vertical plates of MS medium (A) or MS medium in the absence of different nutrient compounds: (B) sulphur, (C) nitrogen and (D) phosphate. Representative images are shown. Scale bars = 1 cm.

## Slide 5
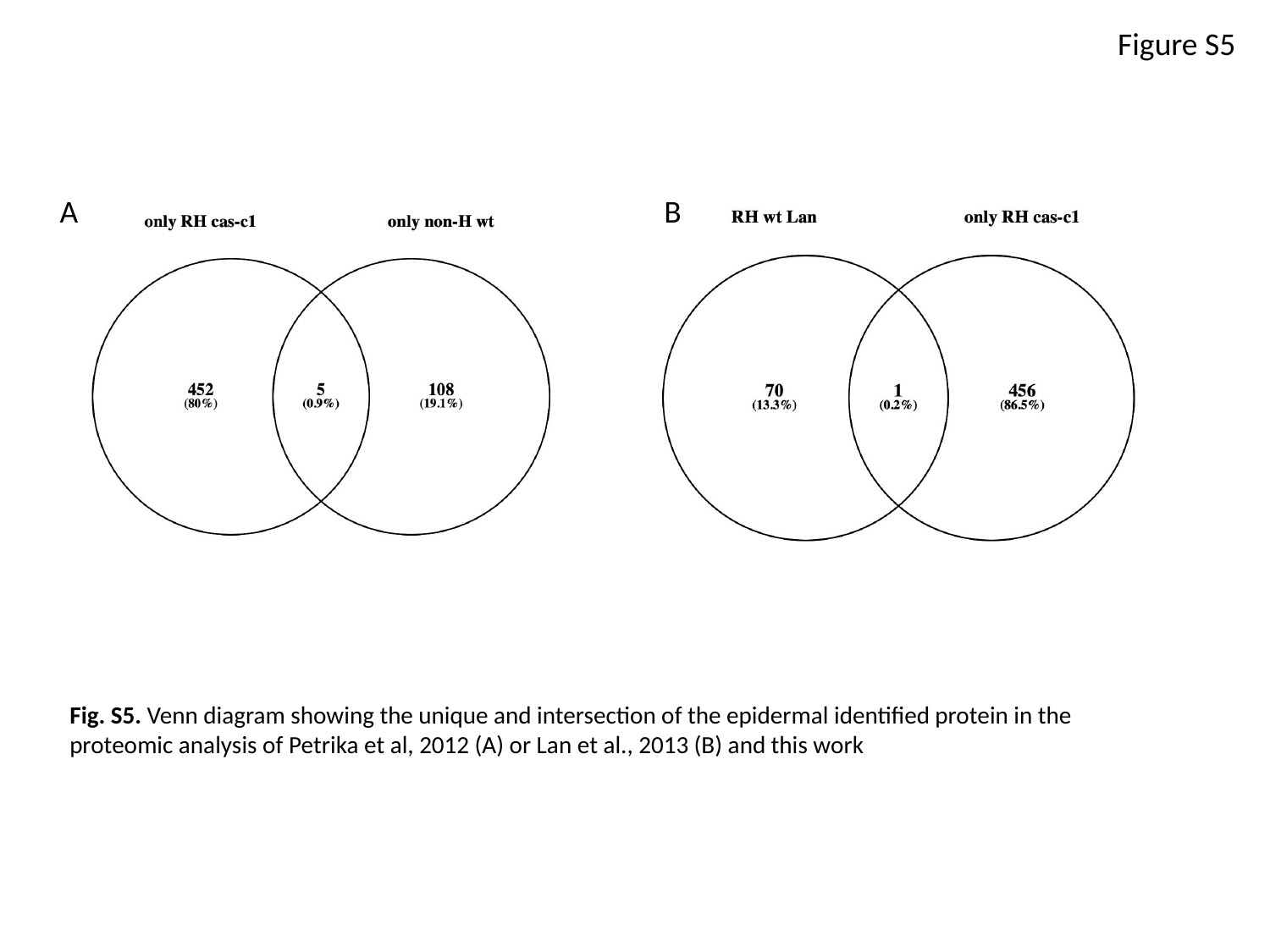

Figure S5
A
B
Fig. S5. Venn diagram showing the unique and intersection of the epidermal identified protein in the proteomic analysis of Petrika et al, 2012 (A) or Lan et al., 2013 (B) and this work

## Slide 6
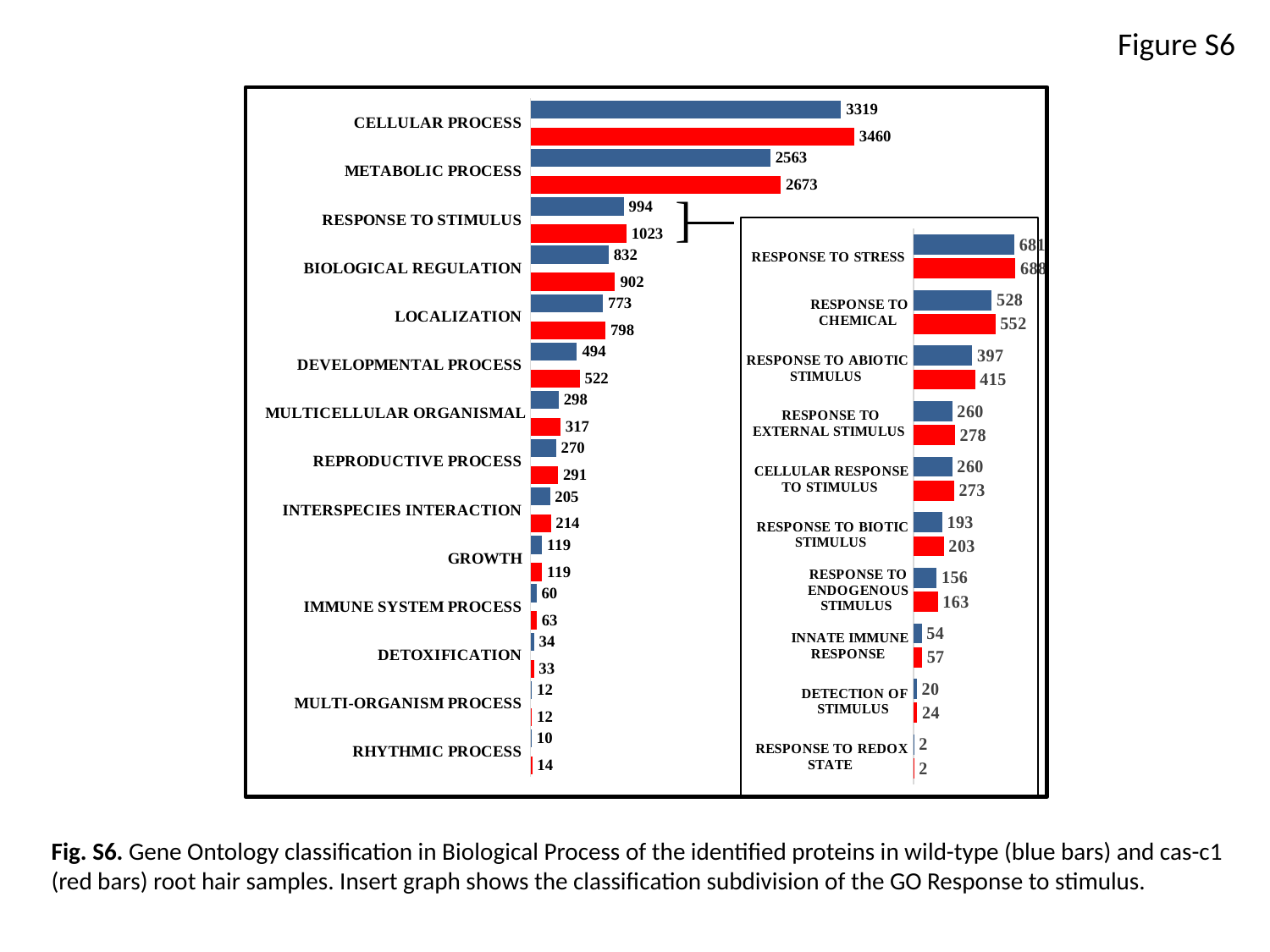

Figure S6
### Chart
| Category | | |
|---|---|---|
| CELLULAR PROCESS | 3319.0 | 3460.0 |
| METABOLIC PROCESS | 2563.0 | 2673.0 |
| RESPONSE TO STIMULUS | 994.0 | 1023.0 |
| BIOLOGICAL REGULATION | 832.0 | 902.0 |
| LOCALIZATION | 773.0 | 798.0 |
| DEVELOPMENTAL PROCESS | 494.0 | 522.0 |
| MULTICELLULAR ORGANISMAL | 298.0 | 317.0 |
| REPRODUCTIVE PROCESS | 270.0 | 291.0 |
| INTERSPECIES INTERACTION | 205.0 | 214.0 |
| GROWTH | 119.0 | 119.0 |
| IMMUNE SYSTEM PROCESS | 60.0 | 63.0 |
| DETOXIFICATION | 34.0 | 33.0 |
| MULTI-ORGANISM PROCESS | 12.0 | 12.0 |
| RHYTHMIC PROCESS | 10.0 | 14.0 |
]
### Chart
| Category | | |
|---|---|---|
| RESPONSE TO STRESS | 681.0 | 688.0 |
| RESPONSE TO CHEMICAL | 528.0 | 552.0 |
| RESPONSE TO ABIOTIC STIMULUS | 397.0 | 415.0 |
| RESPONSE TO EXTERNAL STIMULUS | 260.0 | 278.0 |
| CELLULAR RESPONSE TO STIMULUS | 260.0 | 273.0 |
| RESPONSE TO BIOTIC STIMULUS | 193.0 | 203.0 |
| RESPONSE TO ENDOGENOUS STIMULUS | 156.0 | 163.0 |
| INNATE IMMUNE RESPONSE | 54.0 | 57.0 |
| DETECTION OF STIMULUS | 20.0 | 24.0 |
| RESPONSE TO REDOX STATE | 2.0 | 2.0 |Fig. S6. Gene Ontology classification in Biological Process of the identified proteins in wild-type (blue bars) and cas-c1 (red bars) root hair samples. Insert graph shows the classification subdivision of the GO Response to stimulus.

## Slide 7
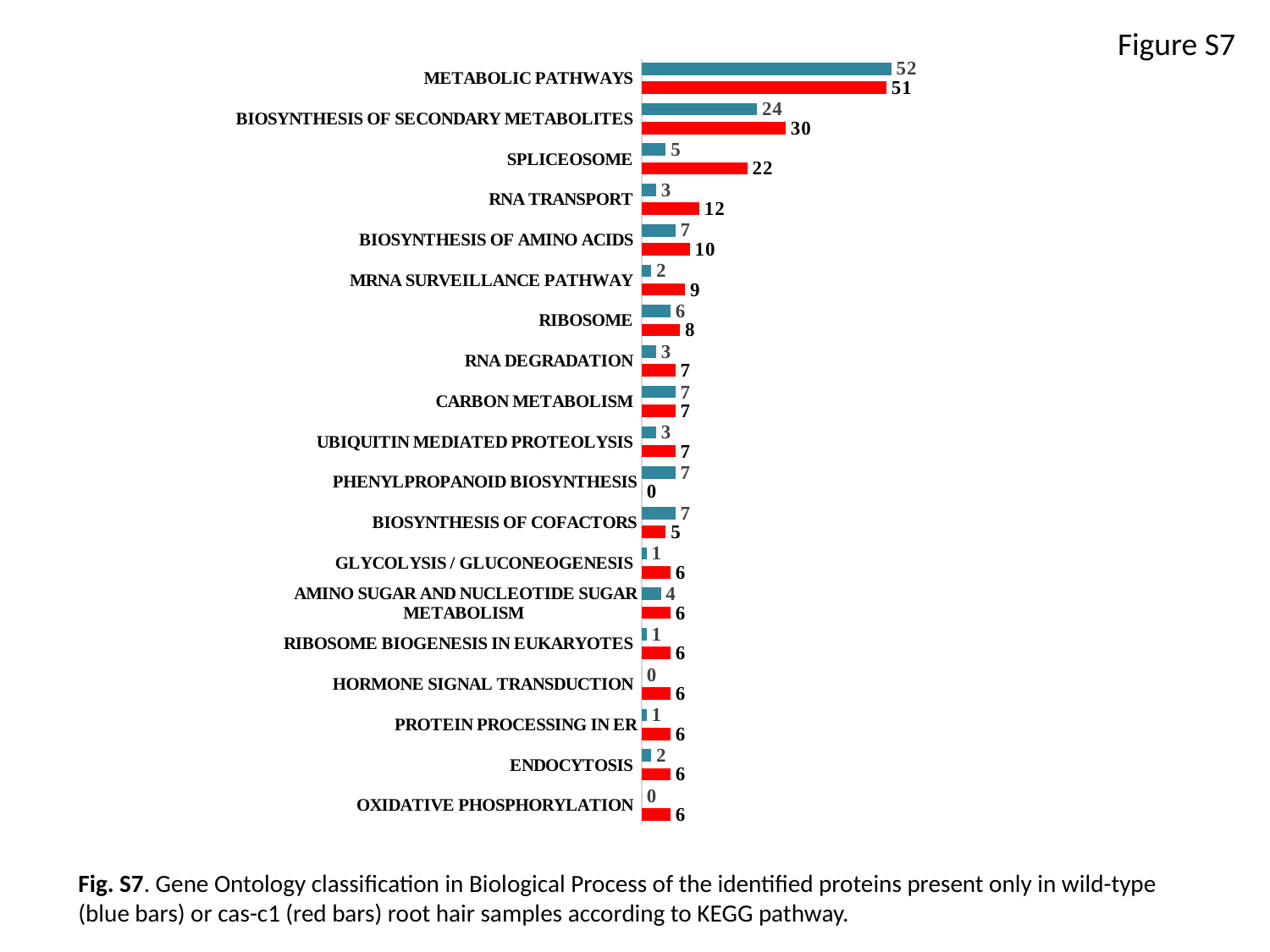

Figure S7
### Chart
| Category | | |
|---|---|---|
| METABOLIC PATHWAYS | 52.0 | 51.0 |
| BIOSYNTHESIS OF SECONDARY METABOLITES | 24.0 | 30.0 |
| SPLICEOSOME | 5.0 | 22.0 |
| RNA TRANSPORT | 3.0 | 12.0 |
| BIOSYNTHESIS OF AMINO ACIDS | 7.0 | 10.0 |
| MRNA SURVEILLANCE PATHWAY | 2.0 | 9.0 |
| RIBOSOME | 6.0 | 8.0 |
| RNA DEGRADATION | 3.0 | 7.0 |
| CARBON METABOLISM | 7.0 | 7.0 |
| UBIQUITIN MEDIATED PROTEOLYSIS | 3.0 | 7.0 |
| PHENYLPROPANOID BIOSYNTHESIS | 7.0 | 0.0 |
| BIOSYNTHESIS OF COFACTORS | 7.0 | 5.0 |
| GLYCOLYSIS / GLUCONEOGENESIS | 1.0 | 6.0 |
| AMINO SUGAR AND NUCLEOTIDE SUGAR METABOLISM | 4.0 | 6.0 |
| RIBOSOME BIOGENESIS IN EUKARYOTES | 1.0 | 6.0 |
| HORMONE SIGNAL TRANSDUCTION | 0.0 | 6.0 |
| PROTEIN PROCESSING IN ER | 1.0 | 6.0 |
| ENDOCYTOSIS | 2.0 | 6.0 |
| OXIDATIVE PHOSPHORYLATION | 0.0 | 6.0 |Fig. S7. Gene Ontology classification in Biological Process of the identified proteins present only in wild-type (blue bars) or cas-c1 (red bars) root hair samples according to KEGG pathway.

## Slide 8
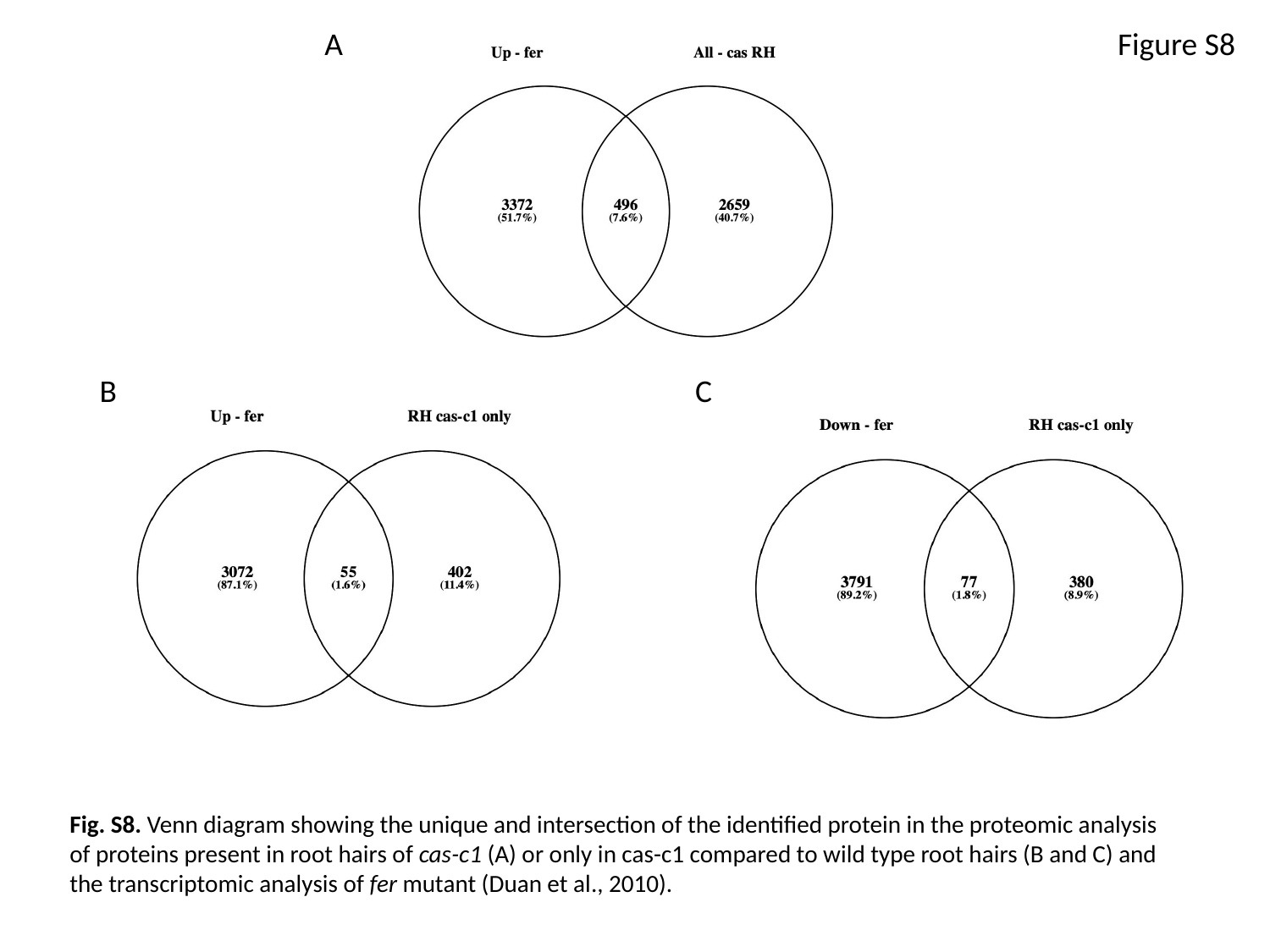

A
Figure S8
B
C
Fig. S8. Venn diagram showing the unique and intersection of the identified protein in the proteomic analysis of proteins present in root hairs of cas-c1 (A) or only in cas-c1 compared to wild type root hairs (B and C) and the transcriptomic analysis of fer mutant (Duan et al., 2010).
